# Terminal microdeletion of chromosome 18 in a Malaysian boy characterized with few features of typical 18q- deletion syndrome: a case report

**DOI:** 10.1186/s13256-023-03984-0

**Published:** 2023-06-10

**Authors:** Azli Ismail, Fadly Ahid, Meow-Keong Thong, Zubaidah Zakaria

**Affiliations:** 1 Haematology Unit, Cancer Research Centre, Institute for Medical Research, National Institutes of Health, Ministry of Health Malaysia, 40170 Shah Alam, Selangor Malaysia; 2 Centre for Medical Laboratory Technology Studies, Faculty of Health Sciences, Universiti Teknologi MARA, 42300 Puncak Alam, Selangor Malaysia; 3 Stem Cell and Regenerative Medicine Research Initiative Group, Universiti Teknologi MARA, 40450 Shah Alam, Selangor Malaysia; 4 Department of Pediatrics, Faculty of Medicine, University of Malaya, 50603 Kuala Lumpur, Malaysia

**Keywords:** Array-CGH, Congenital chromosomal disorder, Dysmorphism, Intellectual disability, 18q- deletion syndrome

## Abstract

**Background:**

The 18q- deletion syndrome is a rare congenital chromosomal disorder caused by a partial deletion of the long arm of chromosome 18. The diagnosis of a patient with this syndrome relies on the family medical history, physical examination, developmental assessment, and cytogenetic findings. However, the phenotype of patients with 18q- deletion syndrome can be highly variable, ranging from almost normal to severe malformations and intellectual disability, and normal cytogenetic findings are common, thus complicating the diagnosis. Interestingly, only few characteristic features of typical 18q- deletion syndrome were found in the patient, despite sharing the same critical region. To our knowledge, this is the first report of a Malaysian individual with 18q- terminal microdeletion diagnosed with microarray-based technology.

**Case presentation:**

Here we report a 16-year-old Malaysian Chinese boy, a product of a non-consanguineous marriage, who presented with intellectual disability, facial dysmorphism, high arched palate, congenital talipes equinovarus (clubfoot), congenital scoliosis, congenital heart defect, and behavioral problems. A routine chromosome analysis on 20 metaphase cells showed a normal 46, XY G-banded karyotype. Array-based comparative genomic hybridization was performed using a commercially available 244 K 60-mer oligonucleotide microarray slide according to the manufacturer’s protocol. This platform allows genome-wide survey and molecular profiling of genomic aberrations with an average resolution of about 10 kB. In addition, multiplex ligation-dependent probe amplification analysis was carried out using SALSA MLPA kit P320 Telomere-13 to confirm the array-based comparative genomic hybridization finding. Array-based comparative genomic hybridization analysis revealed a 7.3 MB terminal deletion involving chromosome band 18q22.3-qter. This finding was confirmed by multiplex ligation-dependent probe amplification, where a deletion of ten probes mapping to the 18q22.3-q23 region was detected, and further multiplex ligation-dependent probe amplification analysis on his parents showed the deletion to be *de novo*.

**Conclusion:**

The findings from this study expand the phenotypic spectrum of the 18q- deletion syndrome by presenting a variation of typical 18q- deletion syndrome features to the literature. In addition, this case report demonstrated the ability of the molecular karyotyping method, such as array-based comparative genomic hybridization, to assist in the diagnosis of cases with a highly variable phenotype and variable aberrations, such as 18q- deletion syndrome.

## Introduction

Intellectual disability or developmental delay is a permanent disability with a major impact on the life of the patient, family, and even on society as a whole. It is often associated with other malformations and dysmorphic features, which are described as being syndromic. However, the etiology of intellectual and developmental impairment remains unidentified in 50% of the patients, despite extensive clinical examinations and laboratory investigations. Genomic imbalances caused by chromosomal aberrations (deletions, duplications, inversions, and rearrangements) represent a major genetic cause of multiple anomaly syndromes that include growth and developmental delay and dysmorphism, which present in 25%–50% of most case series [1]. In addition, environmental factors, including the presence of chronic medical illness, use of steroids, or in-utero exposure to teratogenic agents may increase the chances of chromosomal abnormalities in the offspring [2]. Establishing a diagnosis can be very challenging since the characteristic of many of these syndromes are variable; therefore, it is important to define the broad phenotype and its various causes, including genetic defects to provide better classification, diagnosis, and treatment strategies.

The 18q deletion [Online Mendelian Inheritance in Man (OMIM) #601,808] is a rare congenital chromosomal disorder caused by distal chromosome 18q deletions, with an estimated prevalence of about 1 in 55,000 newborns [3]. The clinical phenotype associated with a terminal deletion of 18q has been well described. It can include cognitive impairment, hypotonia, short stature, ear canal abnormalities, abnormal genitalia, foot deformities, and delayed myelination (that is, dysmyelination) [4]. Other common features include proximally placed thumbs, kidney malformations, and growth hormone deficiency. Dysmorphic features include relative hypertelorism and a flattened midface. However, the limitation of current cytogenetic methods in a clinical setting was unable to provide sufficient resolution to assist in diagnosing the syndrome with high variable phenotype. Here, we report a case of a Malaysian Chinese boy diagnosed to have a terminal deletion of chromosome 18q by array-based comparative genomic hybridization (array-CGH).

## Case presentation

The patient is a 16-year-old Malaysian Chinese boy who presented with multiple problems from early childhood. These included intellectual disability, dysmorphism, delayed milestones, and recurrent infections. His parents were healthy and non-consanguineous. There was no significant family history and problems during pregnancy until birth. He was seen at the University of Malaya Medical Centre at 2 years old for recurrent infections and investigated for immunodeficiency. No definitive diagnosis was made. Physical examination showed he had congenital scoliosis, right congenital talipes equinovarus (clubfoot), right developmental hip dysplasia, cardiac murmur, and behavioral problems. The dysmorphic features included broad forehead, mild hypertelorism, high arched palate, and arm span that was longer than his height. He had a bilateral hearing impairment and hyperactivity. Investigations showed a normal G-banded karyotype, serum amino acid and urinary organic acid and mucopolysaccharidosis screen, fragile X syndrome DNA screening, and thyroid function test. Echocardiogram showed ventricular septal defect and atrial septal defect. A cranial magnetic resonance imaging (MRI) showed no structural abnormalities. His cardiac lesions were corrected by cardiac surgery, and he attended special schooling.

**Fig. 1:**
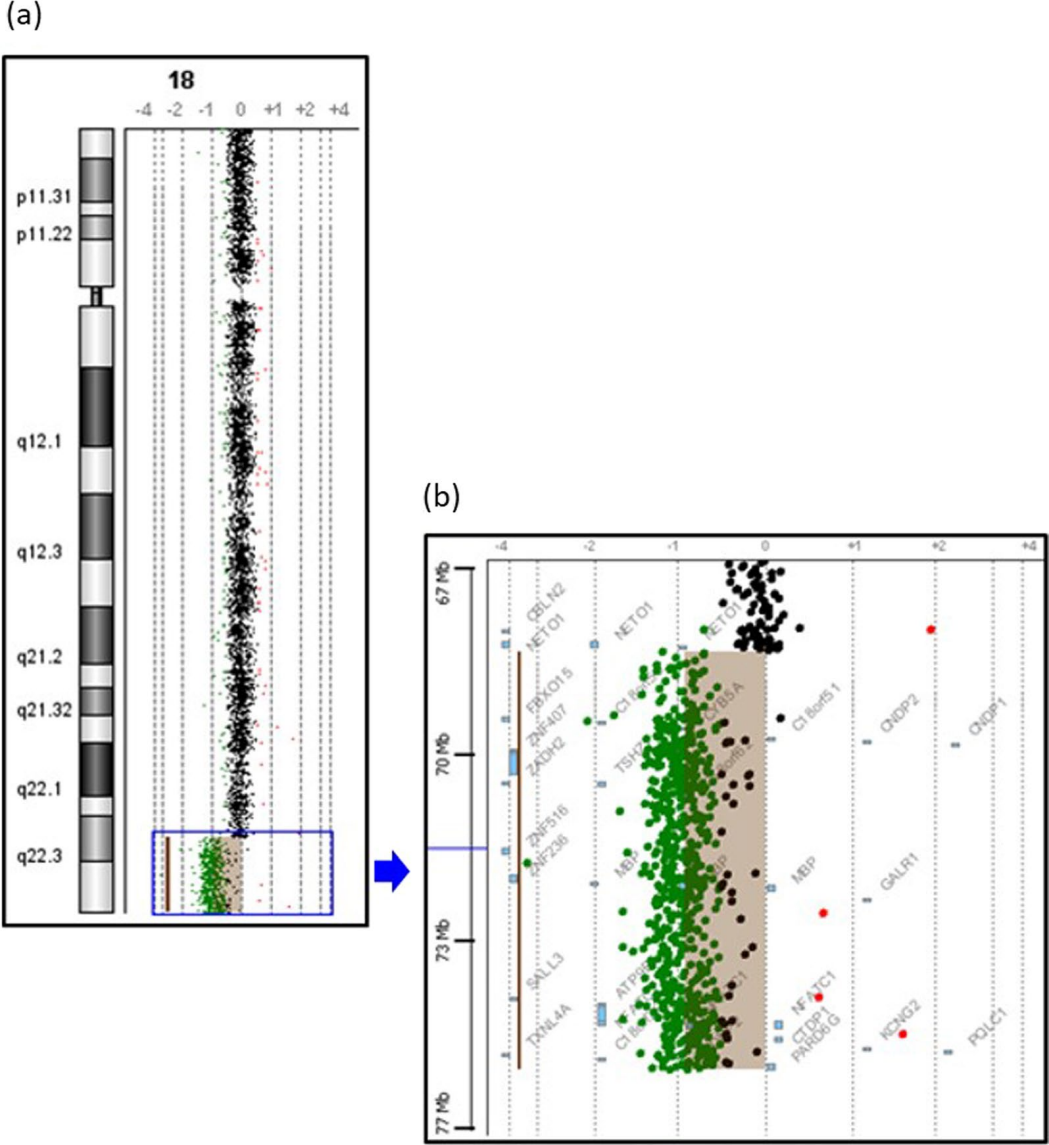
Profile of the microarray analysis showing the deletion region as indicated in the highlighted area. **a** Chromosome view shows a deletion at chromosome 18q22.3-q23. **b** Gene view of the deletion region

**Fig. 2:**
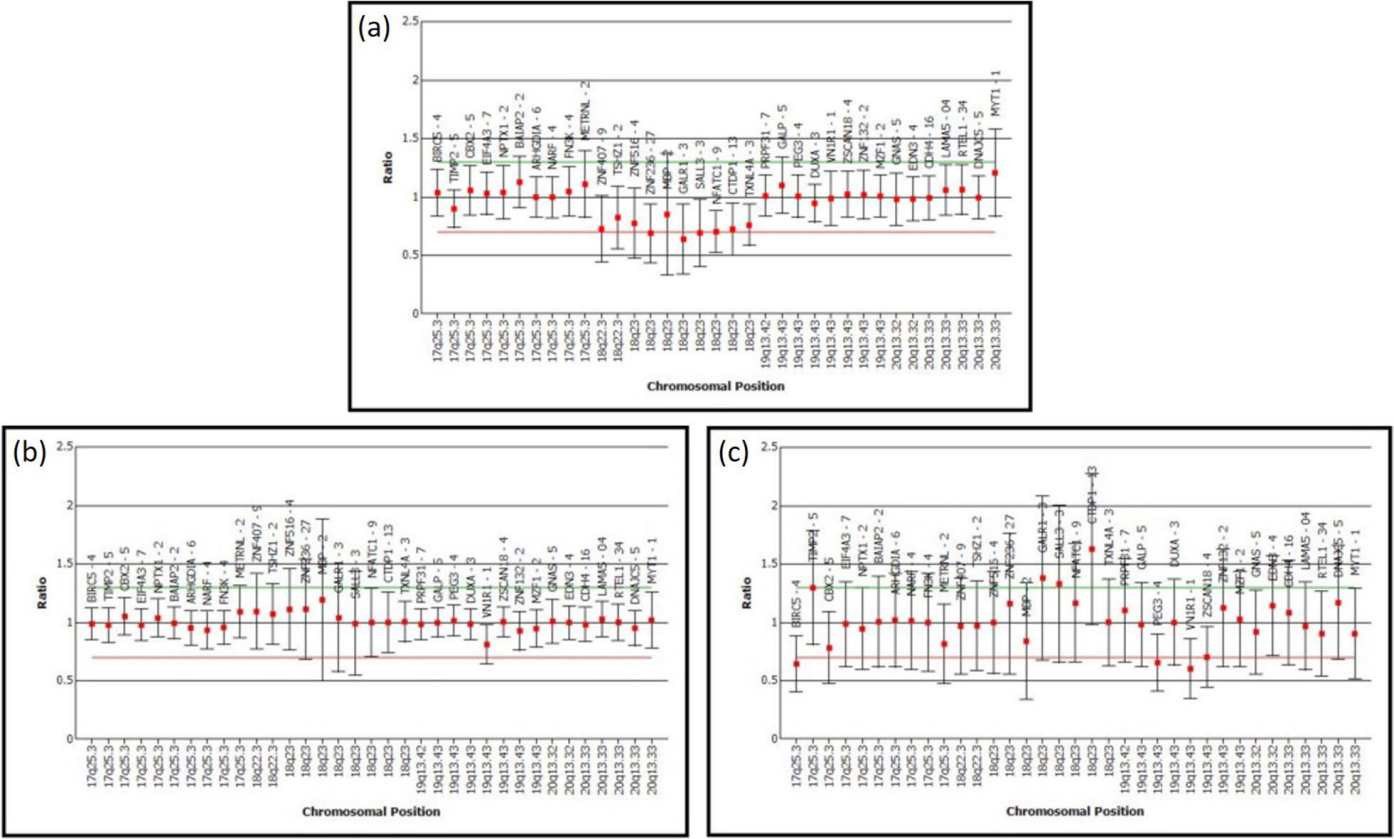
MLPA profile of the patient and his parents. **a** MLPA profile of patient demonstrating a deletion of ten probes mapping to the 18q22.3-q23 region, indicated by cut-off ratio < 0.7. **b** MLPA profile of his father shows no deletion at the 18q22.3-q23 region; **c** MLPA profile of his mother shows no deletion at the 18q22.3-q23 region

Array-CGH analysis at 10 kB resolution (Human Genome CGH Microarray 244A Kit, Agilent Technologies, Santa Clara, CA, USA) revealed a deletion on chromosome 18q22.3-q23, with the first clone locating at 68,755,265 base pairs on proximal 18q22.3 and the last clone locating at 76,111,164 base pairs on distal 18q23, according to UCSC Genome Browser on Human March 2006 Assembly (NCBI36/hg18) (Fig. 1). The size of the deletion was estimated to be about 7.3 MB and encompasses 29 known genes. Multiplex ligation-dependent probe amplification (MLPA) analysis using SALSA MLPA kit P320 Telomere-13 (MRC-Holland, Amsterdam, The Netherlands) showed a deletion of ten probes mapping to the 18q22.3-q23 region, which confirms the array-CGH finding (Fig. 2a), while the parents yielded normal MLPA results, indicating that the deletion occurred *de novo* (Fig. 2b, c).

## Discussion

The 18q- deletion syndrome is a congenital chromosomal disorder resulting from a partial deletion of the long arm of chromosome 18 and represents a contiguous gene deletion syndrome. Like Wolf–Hirschhorn syndrome, Cri-du-Chat syndrome, and Miller–Dieker syndrome, it is a terminal deficiency or deletion syndrome characterized by intellectual disability (formerly known as mental retardation) and congenital malformations. However, the phenotype of patients with 18q- deletion syndrome can be highly variable, thus complicating the diagnosis and becoming a great challenge for both the family and medical professionals, particularly in providing adequate genetic counseling and developing strategies for treatment and prevention. The diagnosis of patients with syndromic conditions such as 18q- deletion syndrome is highly dependent on a comprehensive personal and family medical history, a complete physical examination, and a careful developmental assessment of the child. G-banded karyotyping has been the standard first-tier test for detecting genetic imbalance in the population for more than 50 years; however, this method has limited resolution and is unreliable for the detection of subtle copy number changes. The use of molecular karyotyping methods such as array-CGH enables the detection of submicroscopic chromosomal aberrations at multiple loci and localization of disease gene regions for subsequent candidate gene identification, thus may improve the clinical diagnosis of patients with syndromic conditions.

**Fig. 3:**
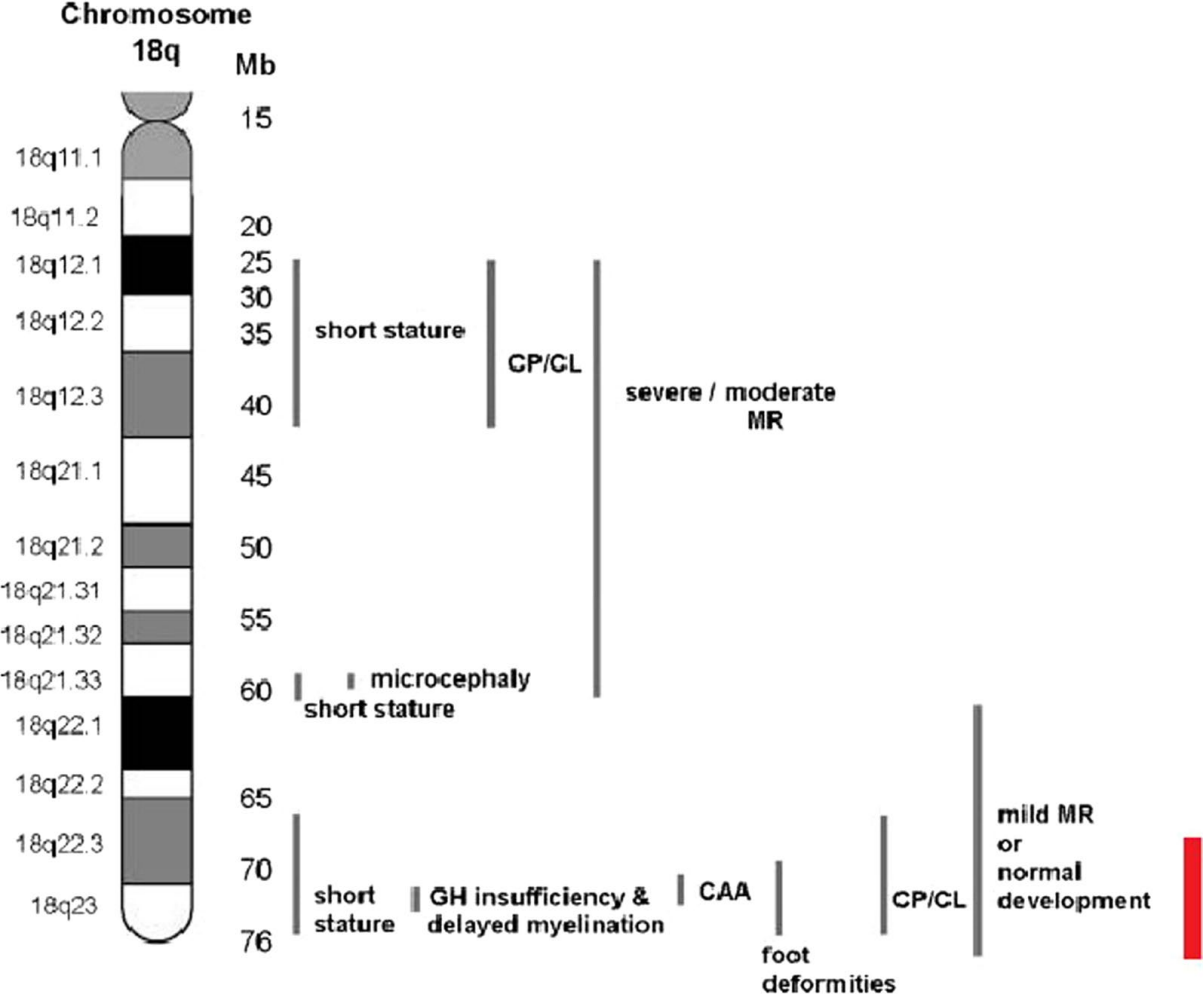
A phenotypic map of chromosome 18q indicating the critical regions for various clinical features. *CAA* congenital aural atresia, *CP/CL* cleft palate/cleft lip, *MR* mental retardation. The deletion presented in our patient is indicated by a red bar. Modified from [7]

The phenotype of 18q- deletion syndrome is highly variable, generally characterized by intellectual disability, microcephaly, short stature, midface hypoplasia, hypotonia, hearing impairment, congenital aural atresia, and foot deformities [4]. Several authors have previously implicated haploinsufficiency of the 18q22.3-q23 region as critical for the 18q- syndrome in relation to white matter abnormalities of the brain, growth hormone deficiency, and congenital aural atresia [4–7]. Cytogenetically, the breakpoint varies greatly, but in most identified cases, the 18q- deletion is terminal and 5–40 MB in size [7]. In another study, high resolution analysis of 18q- using oligo-microarray comparative genomic hybridization demonstrated that the size of terminal deletions varies between individuals from 0.5–30 MB [8]. In addition, several critical regions have been described for specific clinical features (Fig. 3), including microcephaly (18q21.33), short stature (18q12.1-q12.3, 18q21.1-q21.33, and 18q22.3-q23), white matter disorders and delayed myelination (18q22.3-q23), growth hormone insufficiency (18q22.3-q23), and congenital aural atresia (18q22.3) [7]. It is noted that the severity of intellectual disability appears to be milder in patients with deletions distal to 18q21.33 compared with patients with deletions proximal to 18q21.31. Despite having highly variable breakpoints, the critical region for the typical 18q- phenotype has been concluded at 18q22.3-q23, with an estimated size of 9 MB [6]. Table 1 lists the clinical features associated with typical 18q- deletion syndrome [6] and its occurrence in our patient. Interestingly, our patient only presented with few characteristic features of 18q- deletion syndrome. The only distinct characteristic of 18q- deletion syndrome that can be observed in our patient is club feet and hearing loss, despite sharing the same critical region for other clinical features. The clinical presentation of this patient suggests that this patient has a mild form of 18q- deletion syndrome and represents one end of the spectrum of clinical variability seen with 18q terminal deletions, since patients with 18q- deletion syndrome exhibit a wide range of features.

**Table 1:** Summary of clinical findings in our patient with typical 18q- deletion syndrome phenotype

Clinical features	Occurrence
Dysmorphic craniofacial features	
Midface hypoplasia	−
Up/downward slanting palpebral fissures	−
Dysplastic ears	−
Flat philtrum	−
Down-turned corners of mouth	−
Prognathism	−
High or cleft lip/palate	+
Flat nasal bridge	−
Limbs	
Tapered fingers	−
Proximal thumbs	−
Increased whorls on fingers	−
Abnormal toes	−
Club feet	+
Genitourinary	
Hypoplasia of labia/scrotum	−
Central nervous system	
Hypotonia	−
Seizures	−
Hearing loss	+
Strabismus	−
Poor coordination	−
Nystagmus	−
Developmental delay	+
Intellectual disability (formerly known as mental retardation)	+
Delayed myelination	−
Enlarged ventricles	−
Respiratory	
Recurrent respiratory infections	+
Endocrine features	
Growth hormone deficiency	−
Immunology	
Low levels of immunoglobulin A	+

+ Present, − absent

The gene content analysis of the deletion region revealed the presence of four genes that may be indicated as good candidates in generating both neurological and dysmorphic features in the patient. The genes are *TSHZ1*, *CTDP1*, *MBP*, and *GALR1*. The *TSHZ1* gene (OMIM#614427) is teashirt family zinc finger 1 and involved in the development of mice’s ataxial skeleton, middle ear, and soft palate [9]. Hemizygosity of this gene has been reported as a possible candidate gene for congenital aural atresia and external auditory canal aplasia/hypoplasia [10], which may be responsible for the bilateral hearing loss of our patient. *CTDP*1 gene (OMIM#604927) has been associated with congenital cataracts, facial dysmorphism, and neuropathy [11]. Feeding, learning and memory, seizures, pain, anxiety, and mood problems have all been linked to the *GALR1* gene (OMIM#600377), which may explain the behavioral problem of our patient [12]; however, the implications of this gene’s hemizygosity remain unknown. On the other hand, *MBP* gene (OMIM#159430) has been a prominent candidate gene for the dysmyelination phenotype in 18q- deletion syndrome. This gene plays a key role in the compaction of central nervous system myelin [13]. However, no evidence of dysmyelination phenotype is seen in our patient. Given the wide range of phenotypes, there is no proof that a single gene is responsible for all of the features in 18q- deletion syndrome. Therefore, it was noted that all the phenotypic features of 18q- deletion syndrome are caused by contiguous gene deletion in chromosomal region 18q22.3-q23.

## Conclusion

To date, there is very little documented evidence of 18q- deletion syndrome ascertained by microarray analysis in the current clinical diagnostic setting in Malaysia. Our case report expands the phenotypic spectrum of 18q- deletion syndrome by presenting a diverse range of typical 18q- deletion syndrome features. In addition, the findings from the present study have confirmed that the molecular karyotyping method, such as array-CGH technique, is able to detect and identify the submicroscopic chromosomal imbalances that are undetectable by G-banded karyotyping and is mandatory to establish the diagnosis of rare genetic conditions, especially cases with a highly variable phenotype and variable aberrations, such as 18q- deletion syndrome.

## Data Availability

The datasets presented in this study are available in the Database of Chromosomal Imbalance and Phenotype in Humans using Ensembl Resources (https://www.deciphergenomics.org/; Patient ID: 254055).

## Abbreviations

Array-CGH: Array-based comparative genomic hybridization
CTDP1: Carboxy-terminal domain phosphatase 1
GALR1: Galanin receptor 1
MBP: Myelin basic protein
MLPA: Multiplex ligation-dependent probe amplification
MRI: Magnetic resonance imaging
OMIM: Online Mendelian Inheritance in Man
TSHZ1: Teashirt Zinc Finger Homeobox 1
